# Shared Genetic and Regulatory Architecture of Congenital Heart Disease and Autism Spectrum Disorder Revealed by Cross-Tissue Multi-Omic Integration

**DOI:** 10.3390/genes17101180

**Published:** 2026-09-24

**Authors:** Min Zhu, Jingkun Fred Zhang, Stella X. Hu, Xuechen Wang, Weize Xu

**Affiliations:** 1 Department of Colorectal Surgery and Oncology and School of Brain Science and Brain Medicine, The Second Affiliated Hospital, Zhejiang University School of Medicine, Hangzhou 310009, China; 2 Liangzhu Laboratory, MOE Frontier Science Center for Brain Science and Brain-Machine Integration, State Key Laboratory of Brain-Machine Intelligence, Zhejiang University, Hangzhou 311121, China; 3 The Pennington School, 112 W. Delaware Avenue, Pennington, NJ 08534, USA; jingkunzhang@hotmail.com; 4 Trinity College of Arts & Sciences, Duke University, Durham, NC 27708, USA; stellahu.077@gmail.com; 5 Shanghai Key Laboratory of Plant Molecular Sciences, College of Life Sciences, Shanghai Normal University, Shanghai 200234, China; wangxc.bio@outlook.com; 6 Children’s Hospital, Zhejiang University School of Medicine, National Clinical Research Center for Child Health, Hangzhou 310052, China

**Keywords:** autism spectrum disorder, congenital heart disease, shared genetic susceptibility, *CHD7*, *MEF2C*, developmental heart–brain axis

## Abstract

**Background:** Congenital heart disease (CHD) and autism spectrum disorder (ASD) co-occur more frequently than expected, suggesting shared developmental susceptibility. We aimed to identify cross-tissue molecular candidates linking cardiac and neurodevelopmental abnormalities. **Methods:** CHD- and ASD-associated genes were curated from MalaCards and SFARI Gene, and their intersection was defined as the CHD- and ASD-associated gene set (CAGES). Shared gene enrichment was assessed against the human protein-coding genome. CHD and ASD genome-wide association data were integrated with heart and brain cis-expression quantitative trait loci using Bayesian colocalization and summary data-based Mendelian randomization with HEIDI testing. Candidates were further evaluated using bulk transcriptomics, six machine learning feature selection methods, independent cohorts, and scTenifoldKnk virtual knockout analyses in developing human heart and cerebral cortex. **Results:** Seventy-three genes were shared between CHD and ASD. *CHD7* showed suggestive colocalization for CHD and ASD, while lower genetically predicted *CHD7* expression was associated with higher odds of both disorders, with nonsignificant HEIDI tests. *MEF2C* was downregulated in tetralogy of Fallot myocardium and selected by all six ASD machine learning methods, with consistently lower expression in ASD. CAGES-derived signatures discriminated independent CHD, ASD cord blood, and cortical cohorts. *CHD7* virtual knockout analysis identified *MEF2C* as a computationally predicted regulatory candidate in the prenatal brain, particularly in late-fetal excitatory neurons and intermediate progenitor cells, with cell-type-specific predicted effects also observed in the developing heart. **Conclusions:** CHD and ASD share molecular susceptibility involving pleiotropic developmental mechanisms. *CHD7* and *MEF2C* emerge as connected cross-tissue candidates across complementary genetically informed, transcriptomic, and developmental regulatory analyses, highlighting prenatal neuronal populations and developmental windows for investigating the heart–brain axis.

## 1. Introduction

Congenital heart disease (CHD) and autism spectrum disorder (ASD) are common developmental conditions affecting anatomically distinct but developmentally interconnected organ systems. CHD comprises a heterogeneous group of structural abnormalities of the heart and great vessels arising during embryonic cardiac morphogenesis. A large systematic review and meta-analysis estimated a global CHD birth prevalence of approximately 9.4 per 1000 live births in studies conducted between 2010 and 2017, although reported prevalence varies according to lesion severity, geographic region, and diagnostic ascertainment [1]. According to the Diagnostic and Statistical Manual of Mental Disorders, Fifth Edition (DSM-5) framework, autism spectrum disorder (ASD) is a heterogeneous neurodevelopmental disorder characterized by persistent deficits in social communication and social interaction, together with restricted and repetitive patterns of behavior, interests, or activities [2]. A systematic review of studies published since 2012 reported a median global autism prevalence of approximately 100 per 10,000 individuals, with substantial variation across populations and ascertainment strategies [3]. Despite their markedly different clinical manifestations, accumulating epidemiological evidence indicates that CHD and ASD co-occur more frequently than expected by chance. A systematic review and meta-analysis involving more than 250,000 participants found that individuals with CHD had nearly twice the odds of receiving an ASD diagnosis compared with individuals without CHD (odds ratio [OR] = 1.99, 95% confidence interval [CI] 1.77–2.24) [4]. More recent population-based evidence has reinforced this association. In a nationwide Danish cohort of 16,473 children with CHD, the risk of ASD was significantly higher than that in both the general population (adjusted hazard ratio [aHR] = 1.54, 95% CI 1.39–1.70) and unaffected siblings (aHR = 1.37, 95% CI 1.11–1.70) [5]. Consistently, a recent umbrella review identified CHD as a neonatal factor associated with increased odds of ASD (OR = 1.35, 95% CI 1.17–1.52) [6]. These observations are consistent with contemporary clinical guidance recognizing neurodevelopmental disorders as an important long-term morbidity among individuals with CHD. Risk arises from a combination of genetic susceptibility, altered fetal and perinatal development, disease severity, perioperative factors, and other environmental influences [7]. Because CHD is frequently detectable prenatally or shortly after birth, whereas ASD is generally identified later in early childhood, elucidating the biological basis of their co-occurrence may provide an opportunity to identify neurodevelopmental vulnerability before the emergence of a definitive ASD phenotype.

Both CHD and ASD have highly heterogeneous and multifactorial genetic architectures. The genetic basis of CHD encompasses chromosomal abnormalities, copy-number variants (CNVs), rare inherited and de novo variants, and common genetic variation, with implicated genes participating in transcriptional regulation, chromatin remodeling, developmental signaling, ciliary function, and cardiac morphogenesis [8]. Exome-sequencing studies of CHD parent–offspring trios have demonstrated an excess of damaging de novo variants, particularly in individuals with accompanying neurodevelopmental or extracardiac abnormalities, and have implicated genes with important developmental functions in both the heart and brain [9]. Subsequent large-scale analyses have further demonstrated contributions from both inherited and de novo variants and substantial overlap between genes disrupted in CHD and those implicated in neurodevelopmental disorders, including ASD [10]. ASD likewise has a complex genetic architecture involving chromosomal abnormalities and CNVs, rare de novo and inherited sequence variants, and a substantial polygenic contribution from common variants [11,12,13]. These observations support the possibility that neurodevelopmental abnormalities in CHD may not arise solely from altered hemodynamics, perioperative cerebral injury, or other secondary consequences of cardiac disease, but may also reflect pleiotropic disruption of developmental programs shared by the heart and brain.

Recent systems-level studies have further supported this concept. Network-based analyses have identified convergent CHD-ASD risk genes with experimental effects on both cardiac and neural development, while CRISPR interference and large-scale de novo mutation studies have implicated shared developmental processes, including ciliary and cell-cycle regulation [14,15,16]. However, several questions remain unresolved. In particular, it is unclear whether shared CHD-ASD susceptibility is accompanied by concordant genetically regulated gene expression in disease-relevant heart and brain tissues, whether candidate genes show reproducible transcriptional alterations across independent cohorts and tissue sources, and whether putative regulatory relationships are restricted to particular developmental stages or cell populations. Addressing these questions requires integration across genetic, transcriptomic, tissue, and single-cell regulatory levels. Chromatin remodeling and transcriptional regulation provide plausible mechanisms through which genetic perturbations may influence development across both organ systems. CHD7 is an ATP-dependent chromatin-remodeling factor with established roles in neural and cardiac development [17,18,19], whereas MEF2C is an essential developmental transcription factor required for cardiac morphogenesis and is also implicated in severe neurodevelopmental phenotypes, including ASD-related features [20,21]. Notably, loss of *Chd7* in murine cardiopharyngeal mesoderm reduces *Mef2c* expression in the second heart field despite the absence of detectable CHD7 binding at the *Mef2c* locus, suggesting a potentially indirect and context-dependent regulatory relationship [19].

Against this background, we investigated the shared molecular basis of CHD and ASD using an integrative cross-tissue, multi-omic framework. We prioritized *CHD7* and *MEF2C* as convergent candidates and identified a putative *CHD7*–*MEF2C* regulatory relationship that varied by developmental stage and cellular context, with the strongest association during prenatal neurodevelopment. These findings provide a testable framework for a developmental heart–brain axis linking CHD and ASD. Accordingly, the remainder of the manuscript is organized as follows. Section 2 describes the datasets and integrative analytical framework. Section 3 presents the results from genetic, transcriptomic, machine learning, and single-cell analyses. The subsequent sections discuss the biological implications, limitations, and main conclusions of these findings in the context of the developmental heart–brain axis.

## 2. Materials and Methods

An overview of the study design and analytical workflow is presented in Schematic Overview (Figure 1). Briefly, the analytical framework comprised five major stages: (i) curation and functional characterization of genes shared between CHD and ASD; (ii) cross-tissue genetic regulatory prioritization using GWAS-eQTL colocalization and SMR/HEIDI analyses; (iii) transcriptomic characterization and machine learning-based candidate prioritization; (iv) construction and independent validation of gene expression signatures; and (v) developmental and cell-type-specific regulatory inference using single-cell virtual knockout analysis.

### 2.1. Curation of CHD and ASD Gene Sets and Evidence Annotation

CHD- and ASD-associated genes were systematically curated from MalaCards and SFARI Gene, respectively. For CHD, we first defined the relevant phenotype space from the ICD-10 Q20–Q28 block (“Congenital malformations of the circulatory system”). Q27 (“Other congenital malformations of peripheral vascular system”) and Q28 (“Other congenital malformations of circulatory system”) were excluded, resulting in the inclusion of ICD-10 codes Q20–Q26. MalaCards was accessed on 23 August 2024 (build 5.18.0.1084). Individual MalaCards disease entries were mapped to Level-4 ICD-10 codes using the MalaCards disease identifier (MCID) together with the “ICD10 via Orphanet” or “ICD10” annotations provided for each disease entry. Among 53 Level-4 ICD-10 entries within Q20–Q26, 45 mapped to one or more MalaCards disease entries, corresponding to 162 unique MCIDs, whereas eight entries had no directly mapped MCID in the database version used. All genes associated with the eligible MalaCards entries were retained without score-based filtering, yielding 457 CHD-associated genes. For ASD, SFARI Gene was accessed on 23 August 2024, using SFARI Gene Release 2024 Q2. The release contained 1176 genes, all of which were included in the analysis. No filtering was performed according to SFARI Genetic Category, Gene Score, syndromic classification, Eagle score, or number of reports; these variables were retained solely as evidence annotations (Supplementary Table S1).

For the statistical assessment of gene set overlap, 20,076 human protein-coding genes annotated in the *Homo sapiens* GRCh38.p14 reference genome assembly (NCBI RefSeq annotation release RS_2025_08) were used as the reference background universe. Both the CHD- and ASD-associated gene sets were restricted to genes represented in this reference universe before construction of the contingency table for Fisher’s exact test. Accordingly, the enrichment analysis tested whether the observed overlap between the curated CHD- and ASD-associated gene sets exceeded that expected by chance within the human protein-coding gene space.

### 2.2. Pathway Enrichment Analysis

Gene Ontology (GO) and Kyoto Encyclopedia of Genes and Genomes (KEGG) enrichment analyses were performed using R software (R Foundation for Statistical Computing, Vienna, Austria) with the clusterProfiler package (v4.10.1) [22,23]. GO enrichment analysis included all three sub-ontologies: biological process (BP), molecular function (MF), and cellular component (CC). Multiple-testing correction was performed using the Benjamini–Hochberg (BH) procedure, and terms with adjusted *p*-values < 0.05 were considered statistically significant. The distribution of enrichment significance was visualized using a histogram of −log_10_ (adjusted *p*-values).

### 2.3. GWAS and eQTL Datasets for CHD and ASD

For CHD, cis-expression quantitative trait locus (cis-eQTL) summary statistics for heart left ventricular tissue were obtained from the Genotype-Tissue Expression (GTEx) Project version 10 (GTEx V10). The Heart–Left Ventricle cis-eQTL dataset was provided in SMR binary (BESD) format and aligned to the GRCh38/hg38 genome assembly. Corresponding CHD genome-wide association study (GWAS) summary statistics were obtained from FinnGen R13 for the endpoint CONGEN_HEART_ARTER [24], defined as Congenital malformations of the heart and great arteries. After genotype quality control, this dataset included 5502 cases and 494,684 controls (total *N* = 500,186) and was based on the GRCh38/hg38 reference assembly.

For ASD, brain-specific cis-eQTL summary statistics were obtained from the BrainMeta v2 [25] resource through the Yang Lab SMR Data Resource. BrainMeta v2 eQTL mapping was based on RNA-sequencing data from 2865 brain cortex samples derived from 2443 unrelated individuals of European ancestry with genome-wide genotype data. The corresponding cis-eQTL summary statistics were provided in BESD format and mapped to the GRCh37/hg19 genome assembly. ASD GWAS summary statistics were obtained from the 2019 iPSYCH–Psychiatric Genomics Consortium (PGC) ASD GWAS meta-analysis [26]. Specifically, we used the publicly available PGC dataset labeled ‘asd2019’ (November 2017 release). This dataset comprised 18,381 ASD cases and 27,969 controls (total *N* = 46,350) of European ancestry and was based on the GRCh37/hg19 genome assembly.

Accordingly, the CHD analysis integrated GTEx V10 Heart Left Ventricle cis-eQTL data with FinnGen R13 CHD GWAS data on GRCh38, whereas the ASD analysis integrated BrainMeta v2 brain cortex cis-eQTL data with PGC/iPSYCH ASD GWAS data on GRCh37. This phenotype-specific data pairing ensured consistency of genome assembly between the eQTL and GWAS datasets within each analysis.

The adult heart and brain eQTL datasets were used as tissue-relevant resources for genetically regulated expression analyses rather than as direct proxies for fetal regulatory architecture. Their use enabled integration of well-powered cis-eQTL summary statistics with the corresponding disease GWAS datasets. However, because CHD and ASD originate largely during prenatal development, regulatory effects that are restricted to fetal stages, transient progenitor populations, or developmentally specific cellular states may not be captured by these adult-tissue eQTL datasets. The resulting colocalization and SMR findings were therefore interpreted as evidence for genetic regulatory prioritization in disease-relevant tissues, rather than as direct evidence of fetal-stage regulation.

### 2.4. Colocalization Analysis

To assess whether expression quantitative trait loci (eQTLs) and disease-associated loci were driven by a shared causal variant, colocalization analyses were performed using the coloc R package (v5.2.3) [27]. The coloc framework uses a Bayesian approach to estimate posterior probabilities (PPs) for five mutually exclusive hypotheses (H0–H4), including H4, which represents the hypothesis that the eQTL and GWAS signals share a common causal variant. For each gene, SNPs located within a ±1-Mb cis-regulatory window centered on the transcription start site (TSS) were extracted from the corresponding eQTL and GWAS datasets. Colocalization was assessed using the coloc.abf() function with default prior probabilities. The eQTL dataset was specified as a quantitative trait, whereas the GWAS dataset was specified as a case-control trait. Analyses were performed across all 22 autosomes, and chromosome-specific results were subsequently combined into a unified set of gene-level colocalization results. Genes were ranked in descending order according to PP.H4, the posterior probability that the eQTL and GWAS association signals share a causal variant. Colocalization evidence was classified into three tiers: high-confidence colocalization (PP.H4 ≥ 0.70), suggestive colocalization (0.50 ≤ PP.H4 < 0.70), and no evidence of colocalization (PP.H4 < 0.50).

### 2.5. SMR and HEIDI Testing

Summary-data-based Mendelian randomization (SMR) analysis was performed using SMR software (v1.4.2; Yang Lab, Westlake University, Hangzhou, Zhejiang, China) [28]. For each gene, the top associated cis-eQTL, defined as the SNP with the smallest eQTL *p*-value within a ±2 Mb window centered on the transcription start site (TSS), was selected as the instrumental variable. The SMR test estimates the association between genetically predicted gene expression and the trait, with the SMR effect estimate (bSMR) calculated as the ratio of the GWAS effect estimate to the eQTL effect estimate for the top cis-eQTL; the corresponding standard error was derived using the delta method [28]. Statistical significance was defined as *p*SMR < 0.05.

The heterogeneity in dependent instruments (HEIDI) test [28] was subsequently applied to distinguish associations consistent with a single shared causal variant (pleiotropy model) from those potentially attributable to distinct causal variants in linkage disequilibrium (linkage model). A HEIDI *p*-value > 0.01 was considered consistent with the absence of significant heterogeneity and, therefore, supportive of the pleiotropy model.

SMR and HEIDI analyses were performed separately for CHD and ASD. Among genes meeting the above criteria in both analyses, only those showing concordant directions of effect, as indicated by the same sign of bSMR for CHD and ASD, were retained for subsequent analyses.

### 2.6. Transcriptomic Datasets of CHD and ASD Collected from GEO

For CHD, two human myocardial transcriptomic datasets were included. GSE35776 comprised mRNA expression profiles from right ventricular myocardium of 16 infants with nonsyndromic tetralogy of Fallot (TOF) without a 22q11.2 deletion and eight normally developing infant controls. The original dataset additionally contained three fetal heart samples, which were not included in the case-control comparison. Gene expression was profiled using the Affymetrix Human Exon 1.0 ST Array (GPL5175; Affymetrix, Santa Clara, CA, USA) [29]. GSE23959 contained right ventricular myocardial samples from six neonates with hypoplastic left heart syndrome (HLHS) and 10 control myocardial samples obtained from five age-matched newborns with structurally normal hearts, with both right- and left-ventricular samples available for each control donor. Expression profiling was performed using the Affymetrix Human Exon 1.0 ST Array (GPL5188; Affymetrix, Santa Clara, CA, USA) [30].

For ASD, three independent human transcriptomic datasets representing peripheral blood, umbilical cord blood, and brain tissue were included. GSE18123 comprised peripheral-blood gene expression profiles from 170 individuals with ASD and 115 controls. Samples were analyzed using two Affymetrix platforms: GPL570 (Affymetrix Human Genome U133 Plus 2.0 Array; Affymetrix, Santa Clara, CA, USA), comprising 66 ASD cases and 33 controls, and GPL6244 (Affymetrix Human Gene 1.0 ST Array; Affymetrix, Santa Clara, CA, USA), comprising 104 ASD cases and 82 controls [31]. GSE123302, generated from the MARBLES and EARLI prospective high-risk pregnancy cohorts, contained umbilical cord blood transcriptomic profiles measured using the Affymetrix Human Gene 2.0 ST Array (GPL16686; Affymetrix, Santa Clara, CA, USA). A total of 224 offspring were included in the publicly available dataset, comprising 53 ASD, 80 non-typically developing (non-TD), and 91 typically developing (TD) participants based on subsequent developmental outcomes. The subsequent analyses included the 53 ASD and 91 TD samples, while the 80 non-TD samples were not included [32]. GSE102741 consisted of RNA-sequencing data from postmortem dorsolateral prefrontal cortex (DLPFC) tissue from 13 individuals with ASD and 39 matched controls. Libraries were generated using ribosomal RNA depletion and paired-end sequencing on the Illumina HiSeq 2000 (GPL11154; Illumina, Inc., San Diego, CA, USA) platform [33].

### 2.7. Transcriptomic Preprocessing and Harmonization

Raw expression data from each platform were independently processed using background correction, log_2_ transformation, and gene-level summarization. Platform-related batch effects were corrected using ComBat (sva package, v3.50.0) [34] on the merged log_2_-transformed expression matrix, with platform specified as the batch variable and diagnostic group included as a covariate to preserve biological variation. Genes with missing adjusted *p*-values were removed, followed by quantile normalization of the corrected expression matrix. Both the pre-ComBat and fully corrected matrices were retained for comparison. Principal component analysis (PCA) was performed on the normalized gene-level expression matrix using the FactoMineR R package (v2.11) as an unsupervised exploratory analysis to visualize the major sources of transcriptome-wide variation and sample-level structure. For datasets containing samples generated on different platforms, PCA was additionally used to evaluate platform-associated variation before and after batch correction.

### 2.8. Differential Expression Analyses

Differential expression between tetralogy of Fallot (TOF) and control samples in GSE35776 was analyzed using the limma R package [35]. A gene-wise linear model was fitted using lmFit(), with disease status included as the explanatory variable and the control group specified as the reference. Empirical Bayes moderation was subsequently applied using eBayes to obtain moderated *t*-statistics and stabilized variance estimates. For each gene, the log_2_ fold change, moderated *t*-statistic, nominal *p*-value, and Benjamini–Hochberg-adjusted *p*-value were calculated.

### 2.9. Machine Learning Feature Selection

Six complementary machine learning approaches were applied to identify discriminative genes for ASD classification. Bootstrap-aggregated classification and regression trees (Bagged CART) and Random Forest (RF) were implemented within a recursive feature elimination (RFE) framework using the caret R package [36,37]. Candidate subset sizes ranging from 2 to the total number of input genes were evaluated, with predictors iteratively ranked and eliminated according to variable importance. Model performance was assessed by cross-validation, and the subset achieving the highest classification accuracy was retained. For RF-RFE, rfFuncs and 10-fold cross-validation were used. Naive Bayes (NB) feature selection was similarly performed using RFE with nbFuncs from the caret/klaR framework [38]. Candidate subsets were evaluated using 10-fold cross-validation, and the gene subset corresponding to the highest cross-validated accuracy was selected. The Boruta algorithm was implemented using the Boruta R package [39]. Feature importance was evaluated by comparing each predictor with randomly permuted shadow features. Genes with importance significantly greater than the maximum shadow importance (*p* < 0.01) were classified as Confirmed, whereas those with lower importance were classified as Rejected. Tentative features were further resolved using TentativeRoughFix(), and genes ultimately classified as Confirmed were retained. Learning vector quantization (LVQ) was implemented using the caret R package [40]. Models were trained with method = “lvq” using repeated 10-fold cross-validation with three repeats. Predictor variables were scaled prior to model fitting, and variable importance was estimated using varImp(). Genes with importance scores > 0.59 were retained and subsequently intersected with Boruta-confirmed genes to identify more robust candidates. Least Absolute Shrinkage and Selection Operator (LASSO) logistic regression was performed using the glmnet R package [41,42]. Candidate genes were first screened by univariate logistic regression, and genes with *p* < 0.05 were retained. LASSO modeling was then repeated over 1000 iterations using different random seeds. In each iteration, 10-fold cross-validation was performed with cv.glmnet() (family = “binomial”, alpha = 1), and the regularization parameter was selected using the lambda.1se criterion. Genes with non-zero coefficients were retained. The frequency of each gene combination across the 1000 iterations was calculated, and the most frequently observed combination was defined as the final LASSO-selected gene set.

### 2.10. Exploratory Gene Expression Signature Construction and External Evaluation

To evaluate the diagnostic performance of the candidate gene panel, multivariable logistic regression models were separately developed for CHD and ASD in their respective training cohorts and independently evaluated in external validation cohorts. Only genes available in both the training and validation datasets were included during model development, ensuring that the identical gene panel and training-derived coefficients could be applied to the external validation cohort.

Given the limited size of the CHD discovery cohort (16 TOF cases and 8 controls), the CHD analysis was prespecified as an exploratory multigene expression signature analysis rather than the development of a clinically stable prediction model. No additional data-driven feature selection procedure was performed within this cohort, because further feature selection or model tuning using the same 24 samples could introduce additional selection-induced overfitting. Accordingly, the discovery cohort performance was treated as apparent in-sample discrimination, and greater emphasis was placed on evaluation of the locked signature in the independent validation cohort.

Logistic regression model and gene signature score

All candidate genes were simultaneously entered into a multivariable logistic regression model using the glm() function with a binomial distribution and logit link:

$$\mathrm{logit}\left[P\left(case\right)\right]=\beta_{0}+\sum_{i=1}^{k}\beta_{i}X_{i},$$

where *X*_i_ represents the expression level of gene *i*, *β_i_* denotes the corresponding regression coefficient, and k is the total number of candidate genes. A composite gene signature score was calculated for each sample using the coefficients estimated in the training cohort:

$$S_{j}=\sum_{i=1}^{k}{\hat{\beta }}_{i}X_{ij},$$

where *S_j_* represents the signature score for sample *j* and *X_ij_* denotes the expression level of gene *i* in sample *j*.

Model discrimination was assessed by receiver operating characteristic (ROC) curve analysis using the pROC R package. The area under the ROC curve (AUC) and corresponding 95% confidence interval (CI) were calculated to quantify the ability of the model to distinguish cases from controls. Model calibration was evaluated using the rms R package. A logistic regression model with the gene signature score as the sole predictor was used to estimate predicted probabilities and construct the nomogram. Agreement between predicted and observed outcomes was assessed using a bootstrap calibration curve with 200 resamples. The Hosmer–Lemeshow goodness-of-fit test [43] was additionally performed using 10 groups, with *p* > 0.05 indicating no evidence of poor calibration. Model concordance was quantified using the C-index derived from Somers’ *D_xy_*.

For external validation, the model developed in the training cohort was locked before application to the independent validation cohort. The gene panel, regression coefficients, and model structure were kept unchanged, with no coefficient re-estimation or model refitting. Gene signature scores and predicted probabilities in the validation cohort were calculated using the training-derived coefficients, and model discrimination and calibration were subsequently evaluated using the same procedures applied in the training cohort.

### 2.11. In Silico Gene Knockout Analysis Using scTenifoldKnk

Virtual single-cell gene knockout analysis was performed using the scTenifoldKnk R package (Cai Lab, Texas A&M University, College Station, TX, USA) [44]. Single-cell gene regulatory networks (scGRNs) were constructed from raw count matrices, and the regulatory consequences of target-gene knockout were evaluated by comparing wild-type (WT) and virtual knockout (KO) networks. Changes in gene connectivity were quantified as fold change (FC), and statistical significance was assessed using Benjamini–Hochberg-adjusted *p*-values. Genes with significant connectivity changes following knockout were defined as differentially regulated genes (DRGs). To assess cell-type-specific effects, scTenifoldKnk was applied independently to each major cell type using the corresponding cell-specific raw count matrix and identical parameter settings. The resulting DRGs were combined across cell types for downstream analyses.

## 3. Results

### 3.1. Identification and Functional Stratification of the Shared CAGES

Following database-specific curation and gene symbol harmonization, the finalized disease-associated gene sets comprised 457 congenital heart disease (CHD)-associated genes derived from MalaCards entries mapped to ICD-10 codes Q20–Q26 and 1176 autism spectrum disorder (ASD)-associated genes from SFARI Gene Release 2024 Q2. Both gene sets were harmonized to a common reference universe of 20,076 human protein-coding genes annotated in the *H. sapiens* GRCh38.p14 reference genome assembly. Their intersection contained 73 genes, which were designated as the CHD- and ASD-associated gene set (CAGES) (Figure 2A; Supplementary Table S1). Using the same 20,076-gene reference universe, the observed overlap was significantly greater than expected by chance, as determined by a one-sided Fisher’s exact test (odds ratio [OR] = 3.18, 95% confidence interval [CI]: 2.46–4.11, *p* = 6.22 × 10^−14^). These findings demonstrate a significant enrichment of shared disease-associated genes between CHD and ASD within a common human protein-coding gene space (Figure 2B).

Gene Ontology (GO) enrichment analysis of the CAGES revealed significant enrichment of biological processes related to muscle and cardiac development, transcriptional and epigenetic regulation, synaptic function, and MAPK/ERK signaling. KEGG analysis similarly identified pathways associated with cardiomyopathy, MAPK and Notch signaling, cell adhesion, stem-cell pluripotency, and long-term potentiation (Figure 2C; Supplementary Table S2). Together, these findings indicate that genes shared between CHD and ASD are preferentially involved in developmental and signaling programs relevant to both cardiac and neurodevelopment, providing functional context for a potential developmental heart–brain connection that warrants further mechanistic investigation.

### 3.2. Colocalization and SMR Analyses Reveal Shared Regulatory Candidate for CAGES

Colocalization analysis provided suggestive evidence that *CHD7* eQTL and GWAS signals colocalized in both disorders, with H4 posterior probabilities of 0.627 for left ventricular eQTL-CHD colocalization and 0.504 for cortical eQTL-ASD colocalization (Figure 3A). Although *CHD7* was not the highest-ranked gene in either analysis, it was consistently prioritized across both disease-specific colocalization analyses.

To further identify genes showing shared regulatory associations across the two disorders, we intersected genes identified by the CHD and ASD SMR analyses and retained those with concordant directions of effect (Figure 3B). This analysis revealed a subset of genes with consistent SMR effect directions across CHD and ASD, among which *CHD7* showed concordant negative associations in both disorders (Figure 3C). Specifically, lower genetically predicted *CHD7* expression was associated with higher odds of CHD (OR = 0.724, 95% CI: 0.536–0.978; *p* = 0.035) and ASD (OR = 0.889, 95% CI: 0.794–0.995; *p* = 0.040). The corresponding HEIDI tests were nonsignificant for CHD (*p* = 0.835) and ASD (*p* = 0.584), indicating no detectable heterogeneity and remaining consistent with a shared causal variant model.

Collectively, the convergent, albeit suggestive, evidence from colocalization and SMR-HEIDI analyses prioritized *CHD7* as a putative shared regulatory candidate for CHD and ASD, suggesting that genetically regulated *CHD7* expression may contribute to the developmental heart–brain axis.

### 3.3. Transcriptomic Characterization and Machine Learning-Based Prioritization of CAGES

To evaluate the transcriptional relevance of CAGES in CHD, we analyzed GSE35776, which included tetralogy of Fallot (TOF), control, and fetal samples. Principal component analysis revealed clear separation among the three groups, indicating distinct global gene expression profiles (Figure 4A). The pronounced separation of the fetal samples is consistent with substantial developmental-stage-related transcriptional variation and was therefore interpreted as developmental context rather than evidence of a CHD-specific effect. The fetal samples were not included in the subsequent TOF versus control differential expression analysis. Among the 73 CAGES genes, differential expression analysis between TOF and control samples identified 24 differentially expressed genes, comprising 12 upregulated and 12 downregulated genes at an adjusted *p*-value of <0.01 (Figure 4A). Their expression patterns across the three groups were further visualized using a hierarchical clustering heatmap (Figure 4B). 

For ASD, we analyzed GSE18123, which comprises samples profiled using two Affymetrix microarray platforms. PCA was therefore first used to examine major sources of global expression variation and to assess the effect of platform-related batch correction (Figure 4C). Following batch correction, samples from the two platforms showed reduced platform-associated structure, whereas ASD and control samples remained substantially overlapping in principal component space. This finding indicates that ASD diagnosis was not a dominant source of transcriptome-wide variation in this peripheral blood dataset and that global expression patterns alone did not provide clear case-control separation. We therefore applied six complementary machine learning algorithms to further prioritize high-confidence ASD-associated candidates from the 73 CAGES genes. These included recursive feature elimination coupled with Bagged Trees, Random Forest, and Naive Bayes (Figure 4D); Boruta and learning vector quantization (LVQ) (Figure 4E); and LASSO regression with repeated 10-fold cross-validation (Figure 4F). Genes selected by at least three of the six algorithms were retained as robust ASD-associated candidates, thereby narrowing the CAGES for subsequent independent external validation. To assess the robustness of this selection criterion, we additionally examined progressively more stringent thresholds of ≥4/6, ≥5/6, and 6/6 algorithms, revealing the corresponding gene intersections (Figure 4G; Supplementary Table S3). Five genes (*UPF2*, *RPS6KA3*, *MEF2C*, *CYFIP1*, and *STAG1*) were consistently selected by all six algorithms, representing the most stable candidates across the different selection thresholds. Their expression patterns were subsequently examined across both microarray platforms (Figure 4H). Notably, among these five consistently prioritized genes, *MEF2C* was the only gene showing lower expression in ASD cases than in controls across both platforms, whereas the other four genes showed higher expression in ASD cases.

### 3.4. Independent External Validation of CAGES-Derived Candidate Genes

To evaluate the reproducibility and cross-cohort discriminatory performance of the transcriptome-derived candidate genes, we constructed multigene classification signatures in the discovery cohorts and assessed their performance in independent external cohorts. For CHD (Figure 5A), an exploratory gene expression signature was constructed from 12 CAGES genes that were significantly downregulated in the GSE35776 TOF cohort (Figure 5B). The discovery cohort comprised only 16 TOF cases and eight controls, and the 12-gene multivariable model showed an apparent AUC of 1.000 (C-index = 1.00). This perfect in-sample discrimination should not be interpreted as evidence of a stable or perfectly performing diagnostic classifier, because gene selection and coefficient estimation were conducted in the same small cohort, creating a substantial risk of overfitting and unstable coefficient estimates. We therefore regarded the discovery cohort performance as exploratory and placed greater emphasis on independent evaluation. When the locked training-derived signature was applied without coefficient re-estimation to the independent GSE23959 cohort, comprising 6 HLHS cases and 10 controls, it retained discriminatory information (AUC = 0.883, C-index = 0.88). Because the validation cohort represents a distinct congenital heart defect, this result supports partial reproducibility of the CAGES-derived transcriptional signal across CHD phenotypes but does not establish a clinically validated TOF diagnostic model.

For ASD (Figure 5D), a 12-gene signature prioritized by the six-algorithm machine learning framework was developed in the GSE18123 peripheral blood cohort (170 ASD cases and 115 controls) (Figure 4G) and independently evaluated in two datasets from distinct tissue sources. The model achieved an AUC of 0.879 (C-index = 0.88) in the training cohort and maintained comparable performance in GSE123302 umbilical cord blood samples (53 ASD cases and 91 controls; AUC = 0.912, C-index = 0.91) and GSE102741 dorsolateral prefrontal cortex samples (13 ASD cases and 39 controls; AUC = 0.807, C-index = 0.81). No significant lack of calibration was detected across the three cohorts (Hosmer–Lemeshow *p* = 0.166, 0.132, and 0.632, respectively) (Figure 5E). Collectively, the consistent performance across independent cohorts and distinct tissue sources supports the reproducibility and generalizability of the CAGES-derived gene signatures for CHD and ASD.

### 3.5. Single-Cell Virtual Knockout Reveals a Developmentally Restricted CHD7–MEF2C Regulatory Relationship in the Heart and Brain

Our preceding population-genetic and transcriptomic analyses independently prioritized *CHD7* and *MEF2C* as key genes potentially linking CHD and ASD. Previous murine studies have shown that loss of *Chd7* reduces *Mef2c* expression in second heart field progenitors, although direct CHD7 binding at the *Mef2c* locus was not detected, suggesting that this regulatory relationship may be indirect [19]. To investigate whether computational perturbation of *CHD7* was associated with altered *MEF2C* regulatory states during human development and whether such associations were cell-type-specific, we performed *CHD7* virtual knockout using scTenifoldKnk in the developing human heart atlas of Farah et al. (2024) [45] and the developing cerebral cortex atlas of Zhu et al. (2023) [46] (Figure 6A).

In the developing heart (9–15 post-conception weeks), *CHD7* virtual knockout at the aggregate level did not identify significant network-level perturbation of *MEF2C* (Figure 6B). In contrast, cell-type-resolved analyses revealed significant *MEF2C* perturbation in neuronal, smooth muscle cell (SMC), and white blood cell (WBC) populations (Figure 6C; Supplementary Table S4). The prominent response of *MEF2C* in neuronal cells is notable given the involvement of neural-related cellular programs in cardiac development, suggesting that the computational association between *CHD7* perturbation and altered *MEF2C* regulatory state may be restricted to specific cellular contexts rather than uniformly detectable across the developing heart.

A stronger developmental dependence was observed in the brain. Early and late fetal samples were grouped as the embryonic stage, whereas infancy through adulthood constituted the postnatal stage (Figure 6E). Following *CHD7* virtual knockout, *MEF2C* was the top-ranked computationally predicted perturbed gene in the embryonic brain, with a network perturbation score reported as FC = 14.3 by scTenifoldKnk (Figure 6D). In contrast, *MEF2C* was not significantly prioritized in the postnatal analysis, suggesting that the computational association between *CHD7* perturbation and altered *MEF2C* regulatory state is developmentally dependent and most prominent during prenatal neurodevelopment. This temporal specificity is consistent with the recognized importance of prenatal cortical developmental programs in ASD susceptibility. Cell-type-specific virtual knockout further localized this computational association to late-fetal excitatory neurons (EN-fetal-late) and intermediate progenitor cells (IPCs), in which *MEF2C* showed significant network perturbation following *CHD7* virtual knockout (Figure 6F; Supplementary Table S4). Prenatal excitatory neuronal lineages and their progenitors represent developmentally vulnerable cellular populations relevant to ASD genetic risk, making the *CHD7*–*MEF2C* perturbation in these populations particularly notable. Collectively, these computational results indicate a developmental and cell-type-specific association between *CHD7* perturbation and altered *MEF2C* regulatory state, with the strongest signal observed during embryonic brain development, providing a potential regulatory connection between the cardiac and neurodevelopmental components of the developmental heart–brain axis. 

## 4. Discussion

In this study, we used an integrative cross-tissue framework to investigate whether CHD and ASD share developmental molecular mechanisms. Evidence from genetic regulatory, transcriptomic, machine learning, and single-cell network analyses converged on regulatory programs relevant to both cardiac and neurodevelopment, with *CHD7* and *MEF2C* emerging as biologically connected candidates through distinct analytical routes. Importantly, this convergence was context-dependent, highlighting developmental timing, tissue specificity, and cell-type-specific regulation as key features of the shared CHD-ASD molecular architecture.

CAGES should be interpreted as a prior knowledge-constrained candidate space rather than an unbiased genome-wide discovery set. CHD-associated genes were derived from MalaCards and defined by ICD-10 Q20–Q26, whereas ASD-associated genes were obtained from SFARI Gene. This design focused subsequent analyses on the intersection between congenital cardiac malformations and neurodevelopmental disorders while limiting the contribution of genes primarily associated with acquired cardiovascular conditions. Thus, the principal value of CAGES lies not in defining 73 entirely novel shared susceptibility genes, but in providing a biologically constrained framework within which candidates could be further prioritized using independent genetic, transcriptomic, and single-cell evidence.

An important limitation of the genetic regulatory analyses is the developmental mismatch between the available eQTL resources and the biological windows most relevant to CHD and ASD. The GTEx left ventricular and BrainMeta cortical eQTL datasets predominantly represent adult tissues and therefore should not be considered proxies for the fetal regulatory landscape. Rather, they provide well-powered, tissue-relevant estimates of genetically regulated expression that can be integrated with available GWAS data. Consequently, the colocalization and SMR analyses can prioritize regulatory candidates whose genetic effects remain detectable in adult cardiac or neural tissues, but they may miss effects that are restricted to fetal development, transient progenitor states, or specific prenatal cell populations. This consideration is particularly relevant to CHD7, a chromatin remodeler whose regulatory activity is highly dependent on developmental and cellular context. The suggestive *CHD7* colocalization signals observed here should therefore not be interpreted as direct evidence of fetal-stage colocalization. Instead, they provide cross-disorder regulatory prioritization that is complemented by our developmental single-cell perturbation analyses, in which the *CHD7*–*MEF2C* association was strongest during prenatal neurodevelopment. Future studies integrating sufficiently powered fetal heart and fetal brain eQTL datasets will be important to determine whether the *CHD7* regulatory signals identified here are preserved, strengthened, or developmentally restricted during the relevant embryonic and fetal stages.

The CHD 12-gene model achieved an AUC of 1.000 in the TOF discovery cohort and 0.883 in an independent HLHS cohort, whereas the ASD 12-gene signature achieved AUCs of 0.879 in adult peripheral blood, 0.912 in umbilical cord blood, and 0.807 in DLPFC. The apparent perfect discrimination of the CHD model should be interpreted cautiously because the GSE35776 discovery cohort included only 24 samples (16 TOF cases and eight controls) for a 12-gene multivariable signature, resulting in substantial risk of coefficient instability, separation, and overfitting. Therefore, the AUC of 1.000 should not be interpreted as evidence of generalizable diagnostic accuracy or clinical utility. Importantly, the locked CHD signature retained discriminatory information in the independent GSE23959 HLHS cohort, suggesting reproducibility of the underlying CHD-associated expression program across cardiac phenotypes rather than definitive validation of a TOF diagnostic classifier. Similarly, the consistent performance of the ASD signature across peripheral blood, umbilical cord blood, and brain tissue suggests that CAGES may capture partially conserved disease-associated expression programs. The cord blood finding is particularly notable because these samples were collected at birth before ASD diagnosis, indicating that part of the CAGES-associated transcriptional signal may be detectable early in life. Larger phenotype-matched cohorts will be required for robust model refinement, validation, and assessment of clinical utility.

The most mechanistically informative observation was the strong developmental dependence of *MEF2C* network perturbation predicted by scTenifoldKnk following *CHD7* virtual knockout. In the developing brain, *MEF2C* was the highest-ranked computationally predicted perturbed gene during the prenatal stage, whereas it was no longer significantly prioritized postnatally. Importantly, scTenifoldKnk represents an in silico perturbation framework based on inferred single-cell gene regulatory networks; therefore, the predicted *CHD7*-associated changes in *MEF2C* regulatory state should not be interpreted as evidence of direct *CHD7*–*MEF2C* regulation. Instead, these findings provide computational support for a testable developmental regulatory relationship. Notably, previous murine studies showed that *Chd7* loss reduces *Mef2c* expression in second heart field progenitors, despite the absence of detectable direct CHD7 binding at the *Mef2c* locus [19]. Together, these findings argue that *CHD7*–*MEF2C* coupling may emerge within specific developmental windows through chromatin context, intermediate transcriptional regulators, enhancer networks, or cell state-dependent regulatory mechanisms.

To further place the computational perturbation results in the context of experimentally observed phenotypes, we compared our findings with curated mouse phenotype resources and previously published loss-of-function models. Mouse Genome Informatics (MGI) documents pleiotropic phenotypes across multiple *Chd7* mutant alleles, involving behavioral/neurological, cardiovascular, growth/size/body, homeostasis/metabolism, and mortality/aging domains [47]. This broad phenotypic spectrum is consistent with the role of CHD7 as a developmental regulator acting across multiple organ systems. Experimental *Chd7* loss-of-function models further support this interpretation: gene trap disruption of *Chd7* causes embryonic lethality in homozygous mice, whereas heterozygous mutants display reduced body size and neurological/vestibular abnormalities [48]; moreover, neural crest-specific deletion of *Chd7* produces severe conotruncal cardiovascular defects and perinatal lethality [49]. In the developing nervous system, *Chd7* deficiency impairs neural stem/progenitor-cell proliferation and neurogenesis, and *Chd7* mutant fetal brains contain fewer intermediate progenitor cells (IPCs) than wild-type brains [50]. The latter observation is particularly notable in relation to our single-cell analysis, in which IPCs were among the prenatal populations showing significant *MEF2C* network perturbation following *CHD7* virtual knockout. The putative *CHD7*–*MEF2C* relationship was also strongly cell-type-dependent. At the aggregate level of the developing heart, cell-type-resolved analyses identified significant *MEF2C* network changes in neuronal, smooth muscle, and white blood cell populations. The *MEF2C* response in cardiac neuronal populations provides an intriguing cellular clue for a developmental heart–brain connection, although it remains hypothesis-generating rather than evidence that cardiac neural cells directly mediate the association between CHD and ASD. In the developing brain, the association was further localized to late-fetal excitatory neurons and intermediate progenitor cells, suggesting that *CHD7* perturbation may alter *MEF2C*-containing regulatory networks within specific prenatal neuronal lineages rather than throughout the brain. These observations support a context-dependent regulatory model in which developmental effects are restricted by tissue, cell type, and developmental stage, such that cell-specific perturbations may be diluted at the bulk-tissue level and may become less detectable in mature tissues.

Notably, *CHD7* perturbation extended beyond *MEF2C* to other CAGES genes with established roles in cardiac and neurodevelopment. In the developing heart, *LAMB1* has been implicated in extracellular matrix-dependent regulation of second heart field deployment [51], *CDH2* is required for N-cadherin-mediated cardiac morphogenesis and has been associated with human congenital heart defects [16,52], whereas *TTN* is essential for embryonic sarcomere maturation and has been linked to congenital structural and contractile cardiac phenotypes [53]. In the developing brain, *FOXP1* has direct human genetic and experimental links to ASD and related neurodevelopmental phenotypes [54,55], while *DSCAM* has been supported by human genetic studies, patient-derived neuronal models, and experimental ASD models [56,57]. *FGF14* has been implicated in ASD-associated genetic variation and neuronal excitability/synaptic transmission [58,59], whereas *SETBP1* is associated with neurodevelopmental phenotypes including ASD features and has been shown to regulate neural progenitor development [60,61]. Together, these findings suggest that *CHD7* perturbation may affect a broader developmental regulatory network encompassing extracellular matrix organization, cell adhesion, contractile architecture, neuronal differentiation, and synaptic function, rather than an isolated *CHD7*–*MEF2C* interaction.

Overall, the principal strength of this study lies in the convergence of complementary multi-omic and multiscale evidence. Both CHD7 and MEF2C are established regulators of cardiac and neural development [18,19,20,62], lending biological plausibility to their convergence in the present analyses. However, interpretation is limited by the developmental mismatch between available eQTL resources and disease-relevant fetal stages, the small CHD transcriptomic cohorts and associated risk of overfitting, and the computational nature of the scTenifoldKnk perturbation analysis. Accordingly, the proposed *CHD7*–*MEF2C* relationship should be regarded as hypothesis-generating and will require validation using fetal tissue-specific regulatory datasets, larger phenotype-matched cohorts, and experimental perturbation models.

## 5. Conclusions

In conclusion, our cross-tissue multi-omic analyses support shared molecular susceptibility between CHD and ASD and prioritize *CHD7* and *MEF2C* as biologically connected candidates. *CHD7* was primarily supported by cross-disorder genetic regulatory evidence, whereas *MEF2C* was independently prioritized through CHD transcriptomic analyses and ASD machine learning approaches. Developmental single-cell virtual knockout analyses further suggested that *CHD7* perturbation is associated with altered *MEF2C* regulatory states in a tissue-, cell-type-, and developmental-stage-dependent manner, with the strongest association observed during prenatal neurodevelopment. Although these findings do not establish a direct causal *CHD7*–*MEF2C* regulatory relationship, they provide a testable molecular framework for shared cardiac and neurodevelopmental susceptibility and identify specific developmental windows and cellular populations for future experimental investigation of the heart–brain axis.

## Figures and Tables

**Figure 1:**
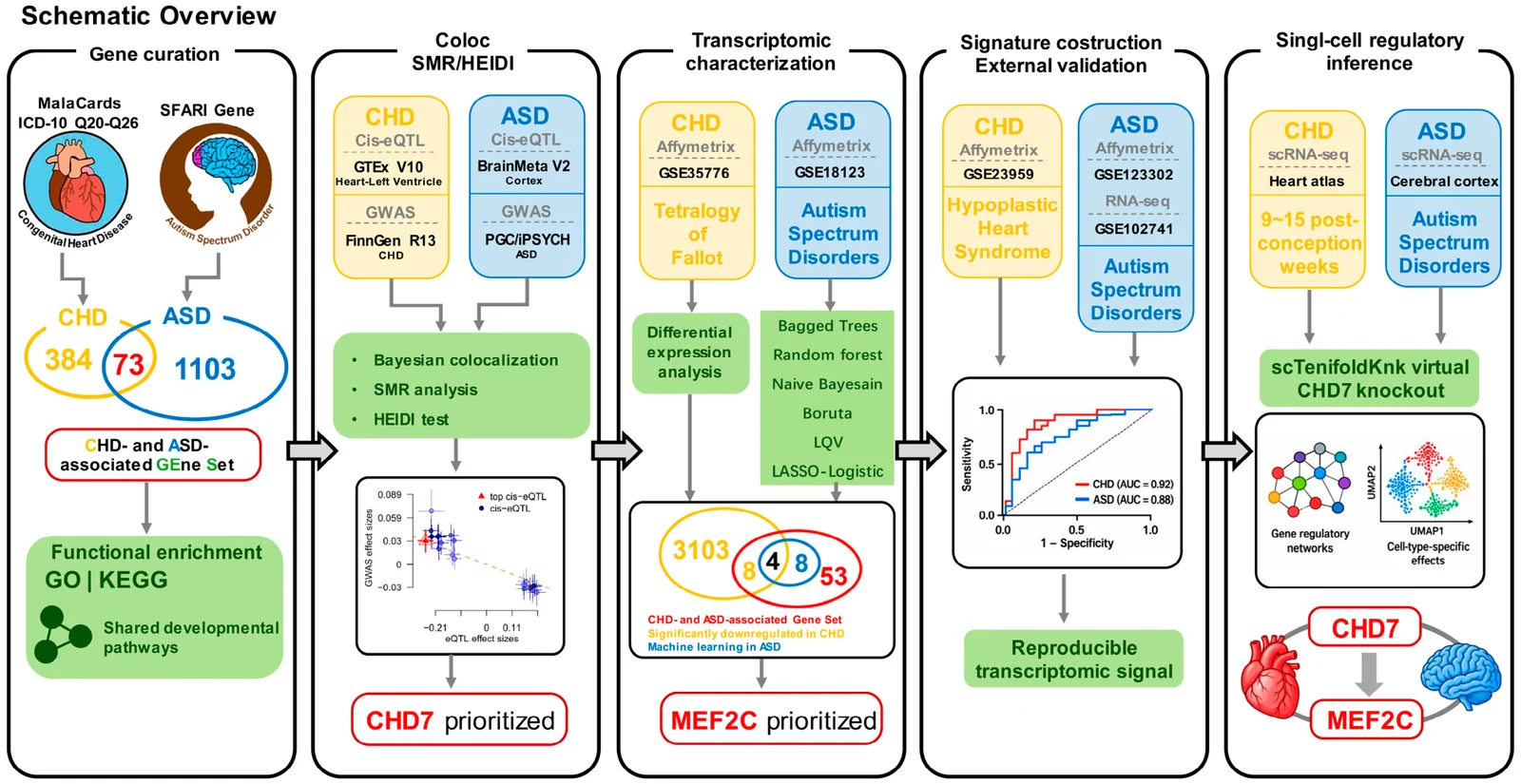
Schematic overview of the study design and analytical workflow.

**Figure 2:**
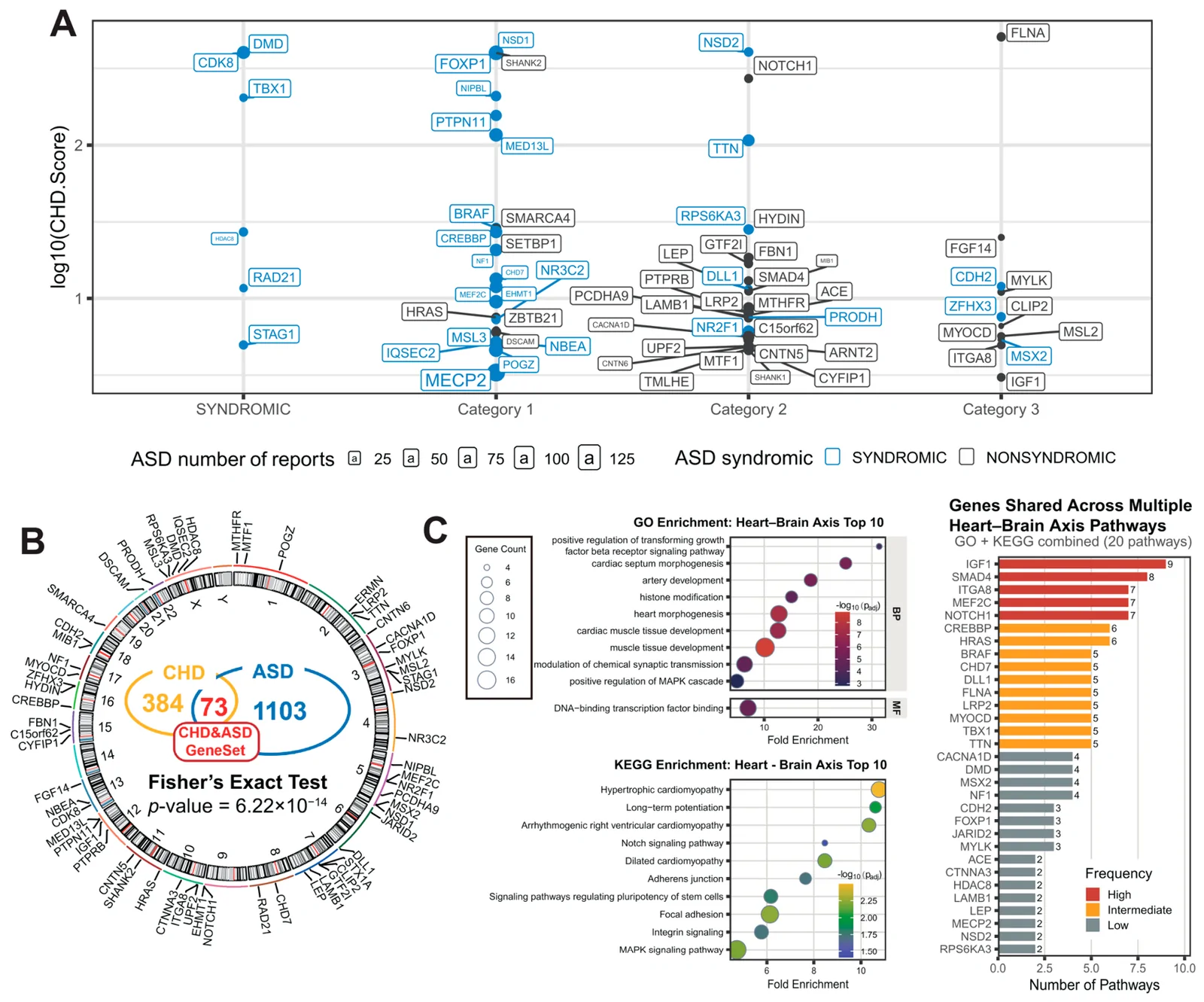
**Identification and functional stratification of shared CSD- and ASD-associated genes.** (**A**) 73 genes shared between CHD and ASD. Shared genes are displayed according to their ASD classification category and CHD association score log10(CHD score). Bubble size represents the number of ASD-related reports for each gene, whereas color denotes whether the gene is annotated as syndromic or nonsyndromic in ASD. (**B**) The shared genes are mapped according to their chromosomal locations around the circular ideogram. The Fisher’s exact test *p* value is also displayed in the figure. (**C**) Functional enrichment and pathway convergence of the 73 shared genes. The upper bubble plot shows the significantly enriched GO terms. The lower bubble plot shows the top enriched KEGG pathways. For both enrichment plots, the x-axis indicates fold enrichment, bubble size represents gene count, and color represents statistical significance as −log10(adjusted *p* value). The bar plot on the right summarizes genes recurrently represented across the combined GO and KEGG heart-brain axis pathways; bar length denotes the number of pathways containing each gene, and colors indicate high, intermediate, or low pathway recurrence frequency.

**Figure 3:**
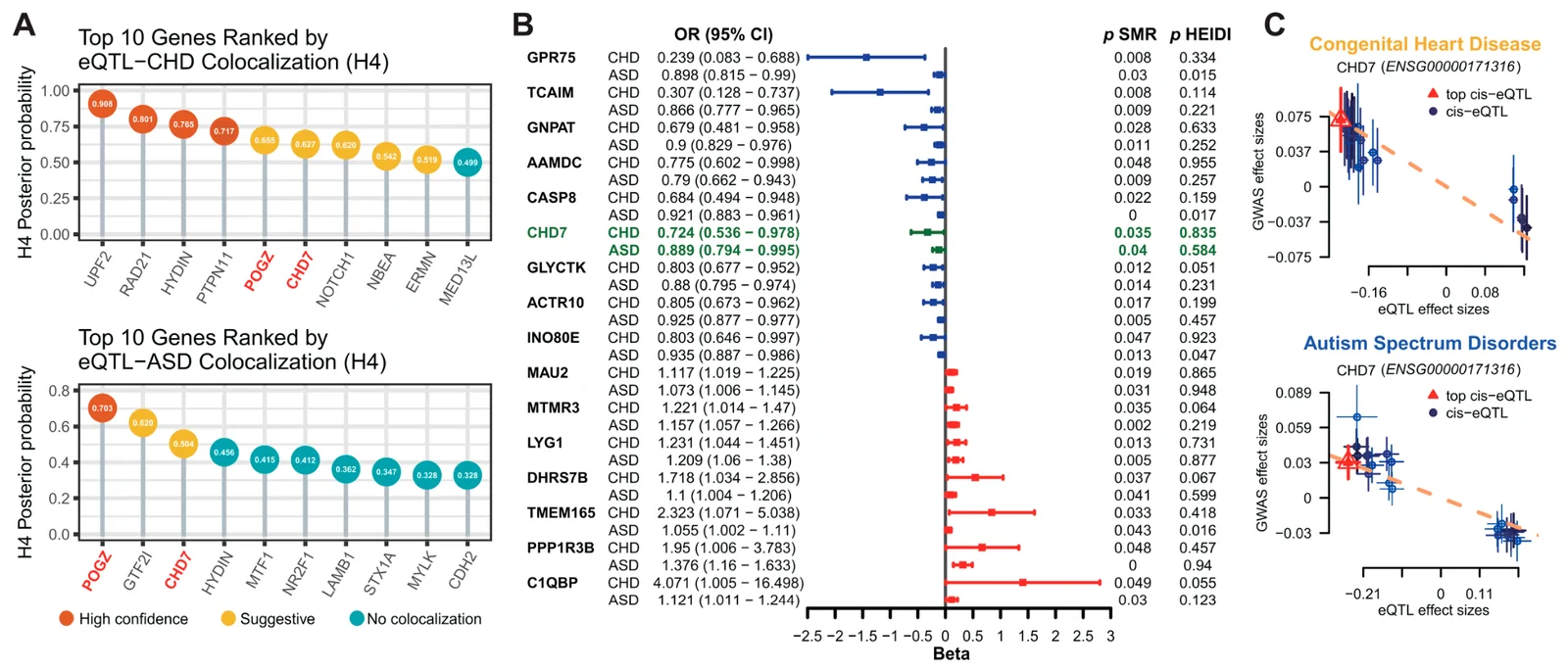
**Colocalization and SMR analyses.** (**A**) Colocalization analysis of CHD and ASD GWAS signals with tissue-relevant eQTLs for candidate genes shared between CHD and ASD. The upper plot shows the top 10 genes ranked by the posterior probability of H4 for CHD, whereas the lower plot shows the corresponding top 10 genes for ASD. Colors indicate the strength of colocalization evidence, categorized as high confidence, suggestive evidence, or no colocalization. (**B**) SMR and HEIDI analyses evaluating associations between genetically predicted gene expression and CHD or ASD. Forest plots show odds ratios (ORs) and 95% confidence intervals (CIs) for each gene-disease association, accompanied by the corresponding SMR (*p* SMR) and HEIDI (*p* HEIDI) test results. CHD7 is highlighted in green. (**C**) Relationship between CHD7 cis-eQTL effect sizes and GWAS effect sizes for CHD (upper) and ASD (lower). Each point represents a cis-eQTL variant for CHD7, with the top cis-eQTL distinguished from the remaining cis-eQTLs.

**Figure 4:**
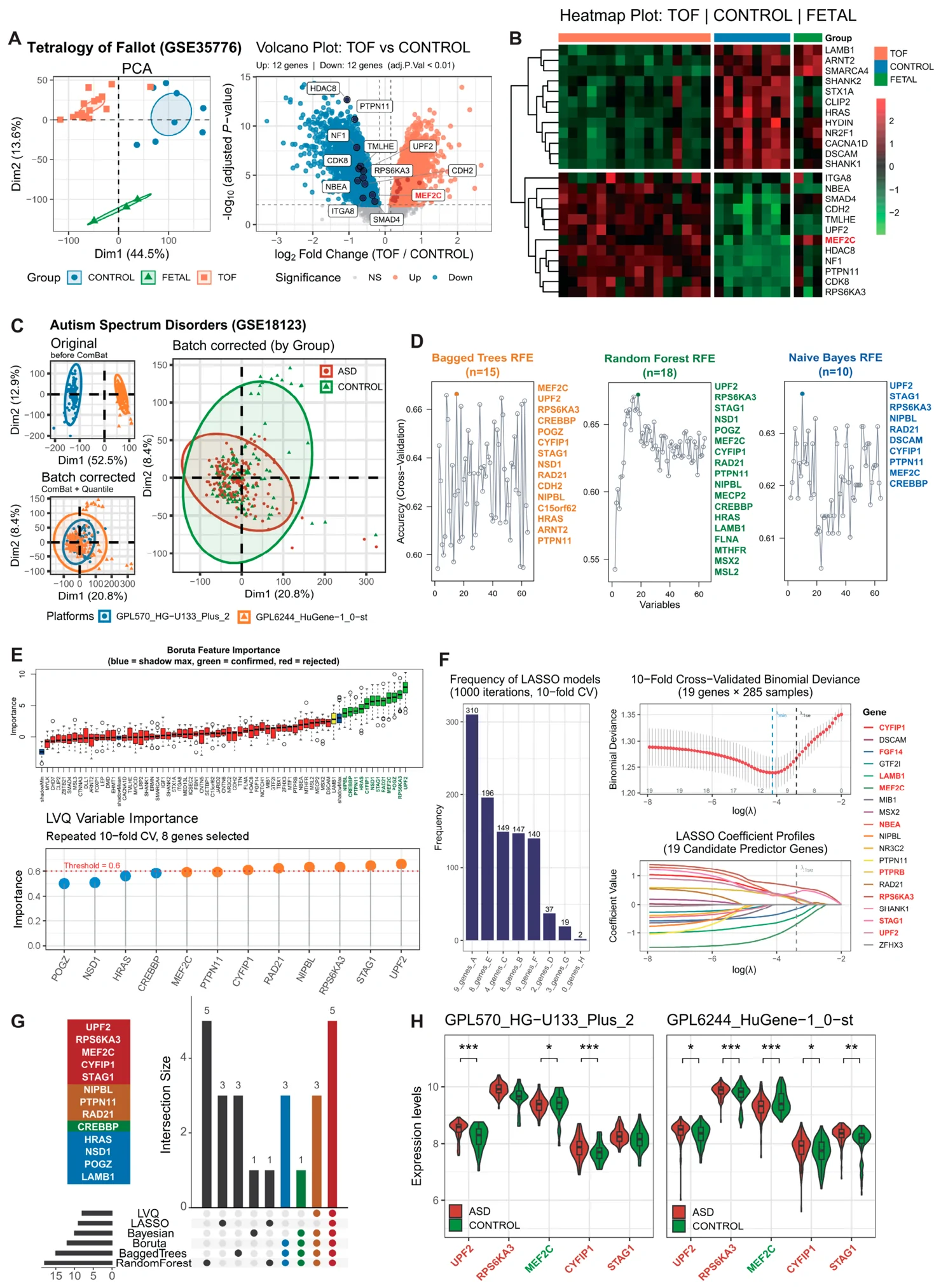
**Transcriptomic characterization and machine learning.** (**A**) Transcriptomic analysis of tetralogy of Fallot (TOF) in GSE35776. Principal component analysis (PCA) shows the distribution of TOF, control, and fetal heart samples. The volcano plot shows differential expression of the 73 CAGES between TOF and control samples. (**B**) Hierarchical clustering heatmap of the 24 differentially expressed genes across TOF, control, and fetal heart samples. Expression values are scaled by gene, with relative high and low expression indicated by the color scale. (**C**) PCA of the GSE18123 ASD transcriptomic dataset before and after batch correction. The left panels show sample distributions according to the two Affymetrix platforms before and after batch correction, and the right panel shows ASD and control samples in the batch-corrected expression space. (**D**) Recursive feature elimination (RFE)-based feature selection using Bagged Trees, Random Forest, and Naive Bayes classifiers. Cross-validated classification accuracy is shown across different numbers of retained predictors, with the optimal subsets containing 15, 18, and 10 genes, respectively. Genes selected by each optimal model are indicated alongside the corresponding plots. (**E**) Feature prioritization using Boruta and learning vector quantization (LVQ). The upper panel shows Boruta feature importance relative to shadow features, with confirmed, rejected, and shadow features indicated as shown. The lower panel shows LVQ variable importance estimated using repeated 10-fold cross-validation; genes exceeding the predefined importance threshold were retained for subsequent integration. (**F**) Least absolute shrinkage and selection operator (LASSO) feature selection. The left panel shows the frequencies of gene combinations identified across 1000 LASSO iterations with 10-fold cross-validation. The upper-right panel shows cross-validated binomial deviance as a function of the regularization parameter λ, and the lower-right panel shows coefficient trajectories for the 19 candidate predictor genes. (**G**) UpSet plot summarizing intersections of genes selected by the six machine learning approaches: Random Forest, Bagged Trees, Boruta, Naive Bayes, LASSO, and LVQ. Genes supported by at least three algorithms were retained as robust candidates. *UPF2*, *RPS6KA3*, *MEF2C*, *CYFIP1*, and *STAG1* were consistently selected by all six approaches. (**H**) Expression distributions of the five genes selected by all six algorithms in ASD and control samples, shown separately for the GPL570_HG-U133_Plus_2 and GPL6244_HuGene-1_0-st platforms. Asterisks denote statistical significance levels as follows: *p* < 0.05 (*), *p* < 0.01 (**), and *p* < 0.001 (***), as indicated in the figure.

**Figure 5:**
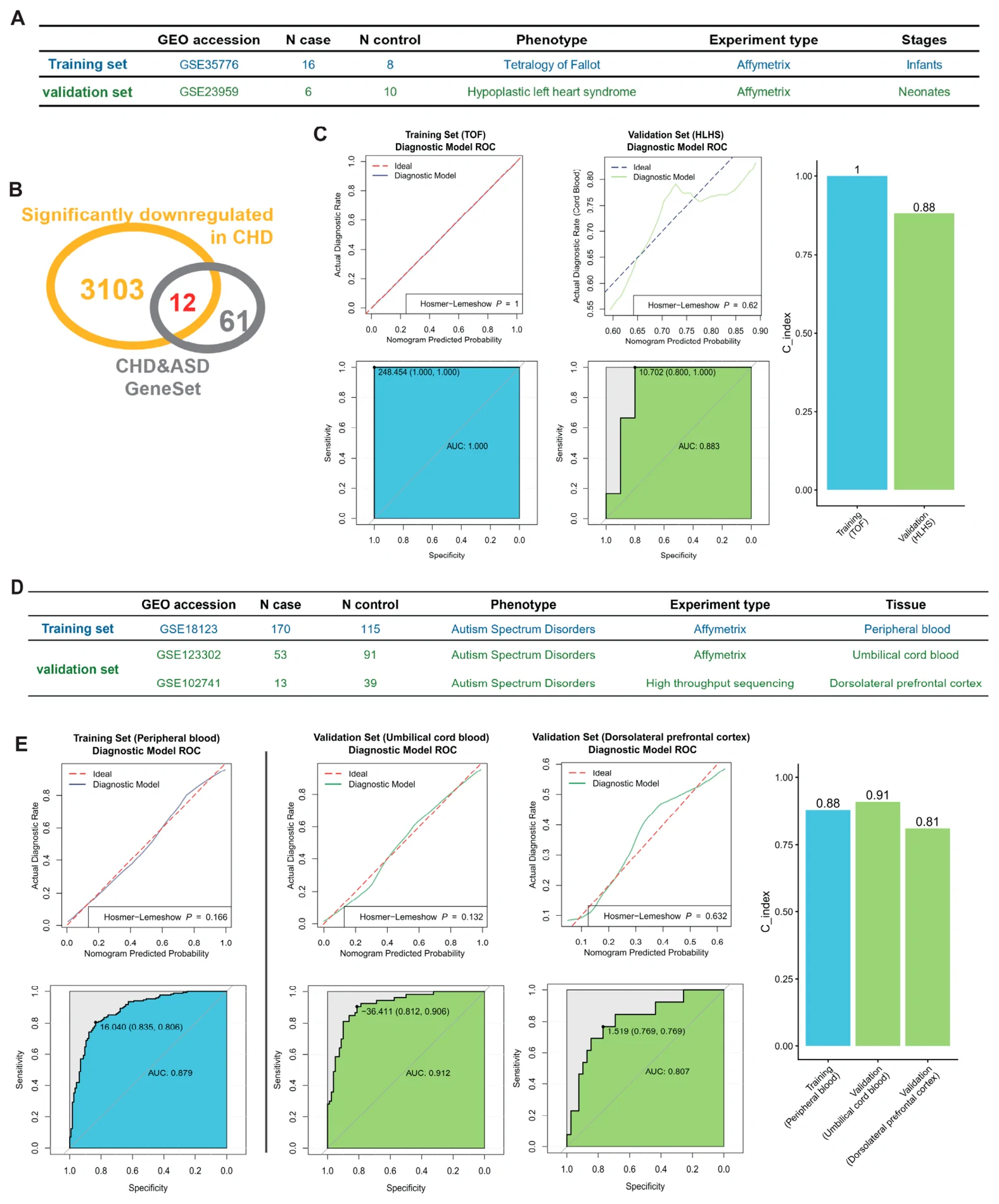
**Independent external validation of CHD and ASD.** (**A**) Characteristics of the congenital heart disease (CHD) discovery and validation cohorts. (**B**) Venn diagram illustrating the derivation of the CHD gene signature. 12 genes were shared between the significantly downregulated genes in the GSE35776 TOF cohort and the CAGES. (**C**) Discrimination and calibration of the CHD gene-expression signature in the discovery and external validation cohorts. Calibration plots compare model-predicted probabilities with observed diagnostic rates in the TOF training cohort and HLHS validation cohort. (**D**) Characteristics of the autism spectrum disorder (ASD) discovery and external validation cohorts. (**E**) Discrimination and calibration of the ASD gene-expression signature across the discovery and external validation cohorts.

**Figure 6:**
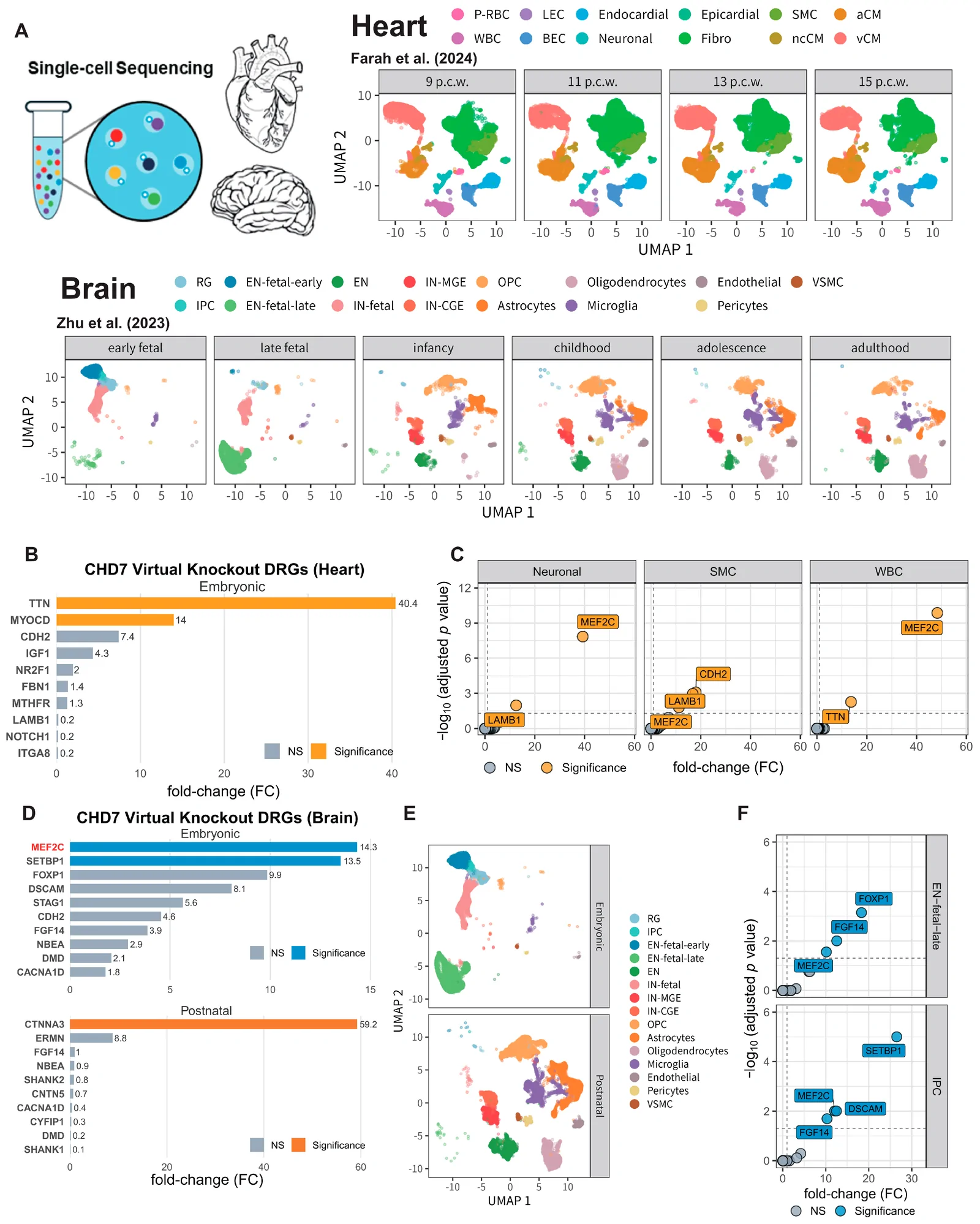
**Single-cell virtual knockout analysis.** (**A**) Overview of the single-cell datasets used for CHD7 virtual knockout analysis. (**B**) Differentially regulated genes (DRGs) following CHD7 virtual knockout in the developing heart at the aggregate embryonic level. Bars indicate Fold change (FC) values, with significance status shown by color. (**C**) Cell-type-resolved CHD7 virtual knockout analysis in the developing heart. Scatter plots show FC versus −log10(adjusted *p* value) for DRGs in neuronal, smooth muscle cell (SMC), and white blood cell (WBC) populations. (**D**) Developmental-stage-specific DRGs following CHD7 virtual knockout in the cerebral cortex. Bars indicate FC values, with significance status shown by color. (**E**) UMAP representation of cerebral cortex cells after grouping samples into embryonic and postnatal developmental stages. (**F**) Cell-type-specific CHD7 virtual knockout analysis in late-fetal excitatory neurons (EN-fetal-late) and intermediate progenitor cells (IPCs). Scatter plots show FC versus -log10(adjusted *p* value) [45,46].

## Data Availability

All data analyzed in this study are publicly available from the following repositories: MalaCards (malacards.org/; accessed on 23 August 2024); SFARI Gene (gene.sfari.org/; accessed on 23 August 2024); GTEx V10 (yanglab.westlake.edu.cn/data/SMR/GTEx_V10_cis_eqtl_summary.html; accessed on 12 March 2025); BrainMeta v2 (yanglab.westlake.edu.cn/data/brainmeta; accessed on 12 March 2025); FinnGen R13 (finngen.fi/; accessed on 12 March 2025); PGC (pgc.unc.edu/for-researchers/download-results/; accessed on 12 March 2025); and GEO (ncbi.nlm.nih.gov/geo/; accessed on 3 December 2025). All data associated with this study are presented within this paper or in the Supplementary Materials.

## Supplementary Materials

The following supporting information can be downloaded at https://www.mdpi.com/article/10.3390/genes17101180/s1. Table S1: Source data and evidence annotation for construction of the CHD-ASD-associated gene set (CAGES), including Sheet 1, MalaCards and SFARI evidence annotations for the 73 shared genes; Sheet 2, the complete SFARI Gene Release 2024 Q2 dataset used in this study (n = 1176 genes); Sheet 3, mapping of ICD-10 Q20–Q26 Level-4 entries to MalaCards disease identifiers (MCIDs). Table S2: Complete Gene Ontology enrichment results, including the biological process (GO:BP), molecular function (GO:MF), and cellular component (GO:CC), together with the KEGG pathway enrichment results for the 73 genes shared between congenital heart disease and autism spectrum disorder. Table S3: Candidate genes identified by six machine learning-based feature selection methods. Table S4: Results of in silico gene knockout analysis using scTenifoldKnk.
